# Detection of myxoma viruses encoding a defective M135R gene from clinical cases of myxomatosis; possible implications for the role of the M135R protein as a virulence factor

**DOI:** 10.1186/1743-422X-7-7

**Published:** 2010-01-16

**Authors:** Graham J Belsham, Charlotta Polacek, Solvej Ø Breum, Lars E Larsen, Anette Bøtner

**Affiliations:** 1 National Veterinary Institute, Technical University of Denmark, Lindholm, 4771 Kalvehave, Denmark; 2 National Veterinary Institute, Technical University of Denmark, Bülowsvej 27, 1790 Copenhagen, Denmark

## Abstract

**Background:**

Myxoma virus is a member of the *Poxviridae *and causes disease in European rabbits. Laboratory confirmation of the clinical disease, which occurs in the autumn of most years in Denmark, has been achieved previously using antigen ELISA and electron microscopy.

**Results:**

An unusually large number of clinically suspected cases of myxomatosis were observed in Denmark during 2007. Myxoma virus DNA was detected, using a new real time PCR assay which targets the M029L gene, in over 70% of the clinical samples submitted for laboratory confirmation. Unexpectedly, further analysis revealed that a high proportion of these viral DNA preparations contained a frame-shift mutation within the M135R gene that has previously been identified as a virulence factor. This frame-shift mutation results in expression of a greatly truncated product. The same frame-shift mutation has also been found recently within an avirulent strain of myxoma virus (6918). However, three other frame-shift mutations found in this strain (in the genes M009L, M036L and M148R) were not shared with the Danish viruses but a single nucleotide deletion in the M138R/M139R intergenic region was a common feature.

**Conclusions:**

It appears that expression of the full-length myxoma virus M135R protein is not required for virulence in rabbits. Hence, the frame-shift mutation in the M135R gene in the nonpathogenic 6918 virus strain is not sufficient to explain the attenuation of this myxoma virus but one/some of the other frame-shift mutations alone or in conjunction with one/some of the thirty two amino acid substitutions must also contribute. The real time PCR assay for myxoma virus is a useful diagnostic tool for laboratory confirmation of suspected cases of myxomatosis.

## Background

Myxomatosis is a disease primarily of European rabbits and is caused by infection with myxoma virus which can result in very high levels of mortality (nearly 100%). In consequence, the virus has been deliberately introduced into some countries as a control measure for wild rabbit populations. Although effective initially, co-evolution of virus and the rabbit population has resulted in the generation of attenuated field strains of the virus and also virus-resistant populations of wild rabbits [1-3].

Infection by myxoma virus of pet rabbits (and presumably wild rabbits too) within Denmark occurs on a regular basis particularly during September and October of each year and it is a notifiable disease. When infection has been suspected clinically by veterinarians, tissue samples from dead or euthanized animals have been submitted to the Danish National Veterinary Institute (DTU Vet) and laboratory diagnosis has been performed using ELISA based systems and/or electron microscopy. The number of cases and suspicions in each year is normally small but, unexpectedly, in 2007 there was a large increase in the number of clinical cases of myxomatosis observed within Denmark (see below and Table 1).

**Table 1:** Analysis of suspect myxomatosis samples submitted for laboratory analysis in Denmark in the period 1998-2007

Year	Number of submissions	Number of positive^a ^submissions (% positive)
2007	195	142 (73%)

2006	3	2 (67%)

2005	3	1 (33%)

2004	4	2 (50%)

2003	7	5 (71%)

2002	3	2 (67)

2001	3	0 (0%)

2000	8	2 (25%)

1999	6	1 (17%)

1998	15	11 (73%)

^a ^Samples were tested by ELISA and/or electron microscopy during the period 1998-2006 (26 of 52 samples proved positive during this period) and in 2007 the samples were analysed using the real time PCR assay described here.

Myxoma virus is a member of the *Leporipoxvirus *genus within the *Poxviridae *(see [3]). The virus has a dsDNA genome of about 160,000 bp and the complete sequence of the virulent Lausanne strain has been determined [4]. The presence of numerous genes encoding immunomodulatory proteins and the narrow host range of the virus has made it an attractive system for analysing the role of these immunomodulators in pathogenesis [3,5]. Poxviruses encode a variety of proteins that block host interferon and tumor necrosis factor mediated antiviral responses. Some of these proteins act within the cell, these include the vaccinia virus E3L and K3L proteins (plus their homologues from other viruses) that inhibit the double-stranded RNA activated protein kinase (PKR)-induced phosphorylation of the eukaryotic translation initiation factor 2 (eIF2) (reviewed in [6]). Other viral proteins act at the cell surface, for example, myxoma virus encodes a protein, called M-T7, which inhibits the interaction of interferon-γ with its cellular receptor [7]. Recently, the product of the myxoma virus M135R gene has been identified as a novel cell surface virulence factor [8]. The M135R protein, which is glycosylated, has some similarity (23% identity) to the vaccinia virus B18R protein which acts as an interferon-α/β receptor. Studies have shown, however, that the M135R protein, which is much shorter than the vaccinia virus B18R product, does not function in this way [8]. However, this same study showed that targeted deletion of the M135R gene (removing 86% of the coding sequence) severely attenuated the virus in European rabbits, with all inoculated animals recovering from the moderate symptoms.

The availability of the myxoma virus genome sequence ([4], [GenBank:NC_001132]) enabled the design of a sensitive and specific real-time PCR-based assay for this virus. This assay has been used to identify the presence of myxoma virus DNA from within laboratory and clinical samples. Furthermore, sequence analysis of selected regions of these myxoma viruses has been performed to explore the relationship between current Danish viruses and other virulent and attenuated virus strains. Surprisingly, a high proportion of the Danish samples, obtained from clinically diseased animals, contained a frame-shift mutation within the M135R gene, which severely truncates the expressed protein product.

## Methods

### Viral DNA isolation

Cell culture grown samples of myxoma virus, vaccinia virus (WR strain), swinepox and Orf virus or rabbit tissue homogenates were used to prepare samples of viral DNA for analysis. The tissues (including nose, lips, eyelids and kidneys) from dead or euthanized pet rabbits which had exhibited clinical disease and had been provided for laboratory diagnosis, were homogenized in physiological saline (1 ml/g of tissue) and then clarified by centrifugation. The DNA was isolated using the QIAamp DNA Blood Mini kit (Qiagen) using the "Blood and Body Fluid Spin protocol" as described by the manufacturer. Briefly, supernatant samples (200 μl) were incubated with Qiagen protease at 56°C for 10 mins, mixed with an equal volume of ethanol and applied to the QIAamp Spin columns. Bound DNA was eluted in water (50 μl) and then assayed directly. It was found that using 1 μl of the eluted DNA was optimal in the real time PCR assay described below, for some samples higher amounts could produce a lower signal, presumably due to the presence of some inhibitor.

In addition, viral DNA was isolated, as described above, from a sample of Cunivak vaccine (supplied from Impfstoffwerk Dessau-Tornau GmbH, Germany) which contains a live attenuated strain of myxoma virus.

### Quantitative real-time PCR detection assay

**Figure 1:**
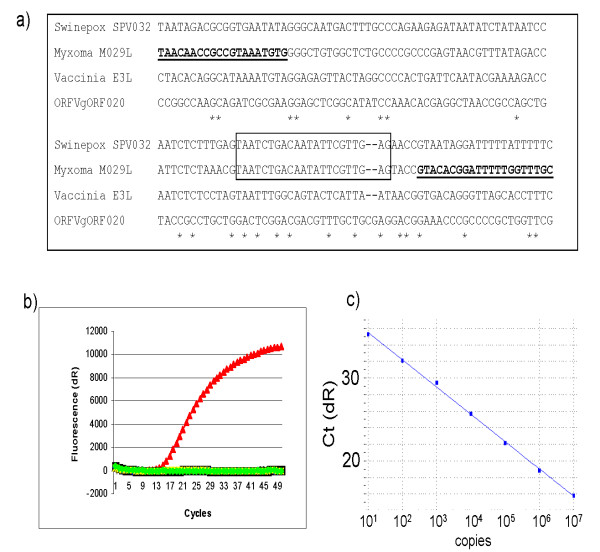
**Design and properties of myxoma virus specific qPCR assay**. **Panel (a)**. The sequence of the myxoma virus M029L gene [GenBank:NC_001132] was aligned with the homologous E3L genes from vaccinia virus (WR strain, [GenBank:AY243312], swinepox [GenBank:NC_003389] and Orf virus [GenBank:NC_005336]) using ClustalW2. The positions of the myxoma virus specific primers (myxE3Lfor and myxE3Lrev) (in bold type and underlined) and the MGB-probe (within the rectangle) which recognizes a sequence that is identical in myxoma virus and swinepox virus are indicated. **Panel (b)**. Viral DNA was prepared from cell culture grown samples of vaccinia virus (WR strain, blue diamond), myxoma virus (red triangle), swinepox virus (black square) and Orf virus (black circle) and each was assayed (in parallel and with negative (water) controls (yellow and green symbols)) in the qPCR assay for myxoma virus as described in Materials and Methods. **Panel (c) **Serial 10-fold dilutions of a plasmid, including the myxoma M029L gene, containing between 10^1 ^and 10^7 ^copies were tested in the myxoma virus assay. The gradient of the line is -3.293 and the efficiency of the PCR reaction was calculated as 101.2%.

The genome sequence of the Lausanne strain of myxoma virus [4] was used as the basis for primer and probe design for a real time PCR assay for detection of the viral DNA. This assay targeted the M029L gene (nt 29307- 28963), which is the homologue of the vaccinia virus E3L gene. The primers (myxE3Lfor with myxE3Lrev) and the MGB probe (SwinemyxE3LCOMP) are listed in Table 2. The PCR was designed to amplify a fragment of 119 bp (see Figure 1a). The assays were performed using the TaqMan Universal Mastermix (Applied Biosystems). Briefly, the samples were treated with uracil-N-glycosylase (UNG) at 50°C for 2 min, followed by incubation at 95°C for 10 min to inactivate the UNG and to activate the TaqGold. Amplification was achieved using 50 cycles of incubation at 95°C for 15 sec followed by incubation at 60°C for 60 sec. Fluorescence was measured after each cycle in a Stratagene MX4000 or MX3005 quantitative PCR thermocycler and the results were analysed using the MxPro software. Primers were purchased from DNA Technology A/S (Risskov, Denmark) while MGB probes were obtained from Applied Biosystems. To assess the sensitivity of the real time PCR assay for myxoma virus DNA, the fragment of the myxoma genome including the entire myxoma virus M029L gene was amplified in a standard PCR using a myxoma virus DNA preparation with the primers MyxM029Lfor and MyxM029Lrev (see Table 2), the product (409 bp) was inserted into pCR-XL-TOPO (Invitrogen) as described by the manufacturer. Plasmid DNA containing the amplified sequence was isolated, quantified (by spectrophotometry) and then a 10-fold dilution series (from 10^7 ^to 10^1 ^copies per μl) was prepared and assayed as above.

**Table 2:** Oligonucleotide primers and probes used in this work

Primers	Sequence (5'-3')
myxE3Lfor	TAACAACCGCCGTAAATGTG
myxE3Lrev	GCAAACCAAAAATCCGTGTAC
MyxM029Lfor	GTTGGTAATTTCATTTCGTACCA
MyxM029Lrev	GGTAAACAATTAATCCGTTGTAA
M135Rfor	CCATACAGAAAGAGATTCAGTTGGA
M135Rrev	GGAGTCTGTATACAGTGATAAATCCTT
M135RXFOR	CCCTCGAGATAAAC***ATG***GTGTTTATATTTATTATC
M135RAPAREV	TTGGGCCC***TTA***CAGGACGCCTAAGGATATC
CATBamfor	TAGGATCCGGG***ATG***GAGAAAAAAATCACTGG
CATXSrev	CCGTCGAC***TTA***CTCGAGCGCCCCGCCCTGCCACTC
M138Lfor	TTAGGAAGATGTCCAGGAATC
M139Rrev	CCGTATTTGTTCGGCAGACC
M153Rfor	GTGTATAGACTGTCGTACCTAC
M154Rrev	ACGGCAATCAACTTCCCGAC
M009Lfor	CAACTGGGATTTCTGCGTAC
M009Lrev	GACGTAATGCGTCGGCGTG
M036Lfor	CAACAGAACGGCAACAATTTATGT
M036Lrev	ACGTGCAGTAACGCCTGTTC
M148Rfor	CGACCATCTGATAATGTATGTC
M148Rrev	ACAGGGATCGAGAGTGAACG
	
**Probe**	
	
SwinemyxE3LCOMP	6FAM-CTCAACGAATATTGTCAGATTA-MGBNFQ

Added restriction enzyme sites within the primer sequences are underlined and nucleotides corresponding to the initiation and termination codons within the M135R and CAT sequences are indicated by bold italics.

### Sequence comparison of the M135R gene from myxoma virus isolates

A standard PCR was performed using the isolated viral DNA preparations from a variety of different myxoma virus samples with the primers M135Rfor and M135Rrev (Table 2) which had been designed to amplify the entire coding sequence of the M135R gene (nt 131699-132235) plus some flanking sequences. The amplicons obtained (653 bp) were purified and sequenced, in both directions, using the same primers on an ABI 3130 automatic sequencer with a BigDye Terminator v3.1 Cycle sequencing kit (Applied Biosystems). The sequences were initially analysed using BioNumerics (Applied-Maths) software and the fasta files were then aligned with the myxoma virus sequence [GenBank:NC_001132] using ClustalW2 [9].

### Expression analysis of M135R proteins

Analysis of M135R protein expression was achieved by making fusion constructs between the coding sequence for chloramphenicol acetyl transferase (CAT) and the M135R coding sequence. The CAT sequence was amplified in a standard PCR using primers CATBamfor and CATXSrev (Table 2) using a derivative of pSV2CAT [10] as template. These primers introduced BamHI, XhoI and SalI restriction sites plus an in-frame termination codon following the XhoI site. The product (739 bp) was inserted into pCR-XL-TOPO (Invitrogen). From the resulting plasmid, BamHI-XhoI and BamHI-SalI fragments were prepared and ligated into a BamHI-XhoI digested derivative of pGEM3Z (Promega) containing a modified polylinker, (T7-BamHI-SmaI-XhoI-EcoRI-ApaI-SalI, as described [11], to produce pGEMCATBS and pGEMCATBX respectively. The former plasmid includes a termination codon at the end of the CAT sequence while the latter does not. The myxoma M135R sequences were amplified from various different myxoma virus DNA samples using the primers M135RXFOR and M135RAPAREV (Table 2) which produced a product of 559 nt including the complete coding sequence of the M135R gene flanked by XhoI and ApaI sites. The products were individually inserted into pCR-XL-TOPO and then XhoI-ApaI fragments were isolated and ligated into similarly digested pGEMCATBX (as described above) to produce a fused open reading frame including the CAT and M135R coding sequences. Structures of these plasmids (see below) were confirmed by enzyme digestion and sequence analysis.

These plasmids were assayed by transfection into BHK cells that had been infected with the recombinant vaccinia virus vTF7-3 [12] essentially as described previously [13] except that FuGene 6 (Roche) was used rather than lipofectin (see [11]). The vaccinia virus vTF7-3 expresses the T7 RNA polymerase and generates capped mRNAs from the transfected plasmids. Cell extracts were prepared 20 h post transfection and analysed by SDS-PAGE and immunoblotting. Expression of CAT and the CAT-M135R fusion proteins was detected using rabbit anti-CAT antibodies (Sigma) and peroxidase labelled anti-rabbit IgG (Dako) with chemiluminescent reagents (ECL Plus, GE Healthcare).

### PCR amplification and sequence analysis of other myxoma virus regions

In addition to the M135R gene, five other regions of the myxoma virus genome were amplified by PCR using primers flanking the insertions and deletions identified within a nonpathogenic myxoma virus strain (6918) by Morales et al. [14]. The primers, listed in Table 2, were designed to amplify a fragment of about 400-500 bp in each case to permit analysis of the M138L/M139R and M153R/M154R intergenic regions plus the coding regions from the M009L, M036L and M148R genes. Amplicons were gel purified and sequenced (by Agowa, Germany) in both directions using the primers used for the PCRs.

## Results

### Design and use of a real time quantitative PCR assay for detection of myxoma virus

A real time quantitative PCR (qPCR) assay to detect specifically myxoma virus DNA was designed. The targeted region of the myxoma virus (a *Leporipoxvirus*) genome together with the homologous sequences from vaccinia virus (*Orthopoxvirus*), swinepox (*Suipoxvirus*) and Orf virus (*Parapoxvirus*) are shown in Figure 1 (panel a). The probe is identical to a region of the myxoma virus M029L gene and also to part of the E3L homolog within swinepox virus (SPV032), however the primer sequences selected to amplify the myxoma virus DNA are very different from the corresponding sequences in swinepox virus. The myxoma probe sequence is significantly different from the corresponding region of the vaccinia virus E3L sequence (7 nt differences out of 22) and from the Orf virus E3L homolog (only 13 nt of the Orf virus sequence match the myxoma virus probe).

To characterize the system for the detection of myxoma virus DNA, viral nucleic acid was prepared from cell culture grown samples of myxoma virus, vaccinia virus, swinepox and Orf virus and tested in the qPCR assay. As expected the assay efficiently detected the myxoma virus DNA but did not produce a signal using nucleic acid preparations from cells infected with the other poxviruses tested (see Figure 1, panel b). As controls, analogous qPCR assays for orthopoxviruses (HA gene, as described previously [15]) and parapoxviruses (B2L gene, encoding the major envelope protein, as described [16]) respectively were also performed. These assays correctly detected their target viruses within these samples, as expected, but did not detect the myxoma virus DNA (data not shown). The qPCR assay for myxoma virus amplified a fragment of 119 bp (including the primer sequences) and this product could be detected by agarose gel electrophoresis at the end of the assay (data not shown) as well as by the change in fluorescence measured in the assay (Figure 1, panel b). To determine the sensitivity of the myxoma virus assay, a region of the myxoma virus genome (409 bp, containing the entire M029L gene) was amplified by PCR from a viral DNA preparation and inserted into a plasmid vector. The resultant purified plasmid was quantified and a dilution series was then prepared and assayed in the qPCR to produce a standard curve (see Fig. 1, panel c). This showed that the assay could detect as little as 10 copies of the myxoma virus sequence in each sample and produced a linear response up to 10^7 ^copies per assay. Thus the assay has a wide range of sensitivity. The specificity of the assay for myxoma virus DNA was confirmed using conventional PCR assays, which targeted other regions of the myxoma virus genome, on DNA preparations from clinical and laboratory samples (see below).

The qPCR assay for myxoma virus has also been applied to 195 tissue samples from dead or euthanized rabbits which had displayed clinical signs of myxomatosis and were submitted by veterinarians to DTU Vet at Lindholm during 2007. From these samples, 142 (73%) proved to be positive (see Table 1) indicating that clinical suspicion had been accurate in most cases. Indeed, most of the clinical samples assayed produced a very high signal (Ct values less than 20). The great increase in the number of samples submitted for laboratory diagnosis is unrelated to the method used for the diagnosis but reflects an exceptional incidence of disease during 2007.

### Comparison of myxoma virus isolates by sequence analysis of the M135R gene

**Figure 2:**
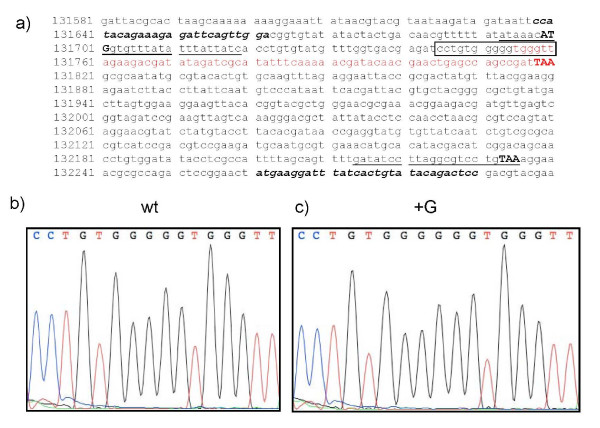
**Evidence for a frame-shift mutation within the M135R gene from Danish samples of myxoma virus**. **Panel (a)**. The nucleotide sequence of the myxoma virus M135R gene, plus flanking sequences, is shown. Primers M135Rfor and M135Rrev (see Table 2), indicated in bold italics, were used to amplify by PCR the M135R gene, together with some flanking sequences at both termini, and these fragments were sequenced directly. The myxoma virus sequences included in primers M135RXFOR and M135RAPAREV (Table 2) for amplifying the coding sequence for M135R are underlined. The initiation and termination codons within these primers are indicated in bold capitals. The region of the gene in which a frame-shift mutation was present in certain virus samples is indicated within a rectangle. **Panels (b) and (c.) **Sequence traces obtained by analysis of a wt M135R gene sequence (as in the myxoma vaccine, panel (b)) and the mutant (+G) form (panel (c)) found in the majority of Danish clinical samples in 2007. The region shown corresponds to the portion of the sequence contained within the rectangle in panel (a). The region of the M135R gene which is predicted to be translated in a different reading frame, downstream of the insertion of a G nucleotide, is indicated in red in panel (a). The termination codons (TAA) for the wt and mutant M135R gene products are indicated in bold capitals.

The region of the myxoma virus M029L gene amplified in the diagnostic qPCR assay is quite short (119 bp). In order to obtain some indication of whether all the cases of myxomatosis in Denmark during 2007 were caused by the same strain of virus, the sequence of a larger region of the genome, from 13 selected samples, was examined. For this purpose, the M135R gene was chosen as a target since it had recently been reported to be an important virulence factor [8]. The DNA preparations from these different clinical samples (which had each been shown to contain high levels of myxoma virus DNA using the qPCR assay described above) plus samples from a myxoma vaccine (Cunivak) and a tissue culture grown sample of myxoma virus were used as the template in standard PCRs to amplify the gene encoding the M135R protein. Each of the tested clinical samples, as well as the vaccine and the cell culture grown virus, generated the expected product in this analysis, supporting the specificity of the qPCR assay which had targeted a completely different region of the genome. The amplified products (ca.650 bp) were then sequenced using the primers used for the PCR reaction (see Figure 2, panel a). The PCR products obtained from the commercial myxoma vaccine and from three of the clinical samples (which each originated from Lolland in south-eastern Denmark, e.g. sample 8770, see Table 3) had sequences which are identical to the published myxoma virus M135R gene sequence [4]. A sample obtained from an archived cell culture grown virus had a single nt substitution (C to T) which encoded a change of amino acid residue 141 from His (H) to Tyr (Y). However, unexpectedly, the ten other recent Danish samples analysed, predominantly obtained from different locations in Eastern Denmark around Copenhagen, each had the same single nucleotide insertion within the M135R coding sequence compared to the published sequence. This mutation resulted in a run of 5 G's being expanded to 6 G's and this insertion was very clear in the original sequence traces (see Figure 2, panels b and c). This sequence change results in a shift in reading frame after the translation of amino acid residue 19 (out of a total of 178 residues for the wt protein). The frame-shift is predicted to result in protein synthesis termination after a total of 40 codons have been translated. The C-terminal region of this aberrant product is predicted to be comprised of amino acid residues decoded in a different reading frame from the wt protein (see Figure 2, panel a).

### Analysis of M135R protein expression from amplified M135R gene sequences

**Figure 3:**
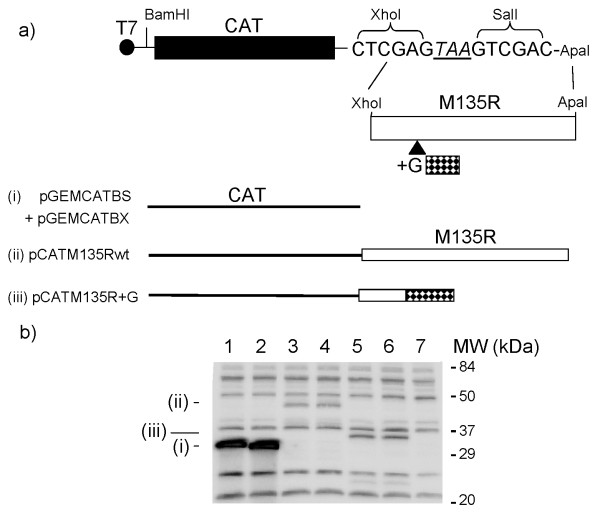
**Effect of the frame-shift mutation on expression of the M135R gene product**. **Panel (a)**. CAT-M135R fusion genes were constructed to demonstrate the effect of the +G insertion within the M135R gene on protein expression. Plasmids containing either the wt (pCATM135Rwt) or the frame-shift variant (pCATM135R+G) of the M135R sequence were constructed. The expected products from the plasmids containing just the CAT open reading frame (ORF) (i), the CAT ORF fused to the wt M135R ORF (ii) and CAT fused to the mutant M135R+G sequence (iii) are illustrated. **Panel (b)**. The plasmids shown in panel (a) were transfected into BHK cells that had been infected with the recombinant vaccinia virus vTF7-3 which expresses the T7 RNA polymerase. Cell extracts were prepared 20 h later and analysed by SDS-PAGE and immunoblotting using rabbit anti-CAT antibodies. Detection was achieved using peroxidase-labelled anti-rabbit IgG and chemiluminescence reagents with a BioRad Chemidoc XRS. Lane 1, pGEMCATBX (without a termination codon at the end of CAT but translation terminates within the vector sequence); lane 2, pGEMCATBS (with a termination codon, see panel (a)); lanes 3 and 4, plasmids (pCATM135Rwt) containing the fused CAT and wt M135R ORFs (n.b., there is no termination codon at the end of the CAT sequence); lanes 5 and 6, plasmids (pCATM135Rwt+G) containing the fused CAT and mutant M135R+G sequences and lane 7, No DNA (negative control). The migration of molecular weight markers is indicated. The products corresponding to those expected from constructs (i), (ii) and (iii) shown in panel (a) are marked.

On the basis of the sequencing data, it was apparent that DNA fragments containing the insertion of a single nucleotide within the M135R coding sequence should encode a very different protein than the wt sequence (see Figure 2, panel a). In order to confirm that this single nucleotide insertion was not a sequencing or PCR artefact, new independent PCR products derived from the viral DNA preparations were made using different primers (M135RXFOR and M135RAPAREV, see Table 2). Sequencing of these new amplicons confirmed the presence of the single G insertion in the majority of the Danish field samples analysed (data not shown). These primers were also designed to allow the fusion of these amplified myxoma virus sequences downstream of the CAT coding sequence (lacking a termination codon, as in pGEMCATBX, see Figure 3) in order to express CAT-M135R fusion proteins.

It was anticipated that the construction of plasmids containing the fused CAT-M135R reading frames should allow the expression of the normal M135R coding sequence to be observed and, more importantly, also the prematurely terminated M135R gene product which by itself would only be 40 amino acids in length, ca. 4.5 kDa, and therefore difficult to detect alone. Different plasmids containing either the wt M135R sequence (e.g. as in the myxoma vaccine) or the frame-shift variant (+G) were constructed and then assayed along with a plasmid encoding the normal CAT coding sequence (see Figure 3). The CAT vectors, pGEMCATBS and pGEMCATBX (both lacking any M135R sequences) very efficiently expressed the CAT protein (about 27 kDa) as expected (Figure 3, panel B). Fusing the wt M135R sequence to the CAT coding sequence (as in plasmid pCATM135Rwt) resulted in the expression of a much larger protein being recognized by the anti-CAT antibodies consistent with the expected fusion product of ca. 45 kDa (Figure 3, panel B, lanes 3 and 4). In contrast, the attachment of the mutant M135R sequences (as in pCATM135R+G), containing the insertion of a single G nt, resulted in a small, albeit readily detectable, decrease in the mobility of the CAT protein (Figure 3, panel B, lanes 5 and 6) consistent with the predicted addition of only an extra 40 amino acid residues (ca. 4.5 kDa) to the C-terminus of CAT. In addition, a smaller, weak band, ca. 22 kDa, was also detected from this plasmid, it seems likely that this is a degradation product derived from the fusion protein which may adopt a partially unfolded structure.

### PCR and sequence analysis of Danish samples of myxoma virus DNA

Very recently Morales et al. [14] have published the complete genome sequence of a nonpathogenic field strain (6918) of myxoma virus isolated in Spain. This sequence was found to be 99.95% identical to the virulent Lausanne strain but the 6918 virus only produces moderate symptoms in inoculated animals and even milder disease in animals infected by contact [17]. In comparison to the parental virus, the 6918 strain had 32 amino substitutions and also four genes were interrupted by frame-shift mutations. Remarkably, one of these frame-shift mutations (in M135R) was identical to the insertion detected in most of the Danish samples analysed here. Thus it was decided to analyse four of the Danish samples, each containing the frame-shift mutation within the M135R gene, for the presence of the other insertions/deletions detected in the nonpathogenic strain 6918. Fragments of ca. 400-500 bp corresponding to five separate regions of the genome including the intergenic regions M138L/M139R and M153R/M154R plus part of the coding regions from the genes M009L, M036L and M148R were amplified by PCR, using primers listed in Table 2, and sequenced. The results are shown in Table 3. We also selected for these analyses one clinical sample (8770) and the Cunivak vaccine strain, which had the wt M135R gene sequence. From the thirty new PCR reactions, the expected products were obtained in all but two cases, no products were detected using the Cunivak DNA with the primers targeting the genes M009L and M036L, presumably sequence differences were present at the primer binding sites. For the samples containing the single nucleotide insertion within the M135R gene, the sequences of the M009L, M036L, M148R and M153R/M154R fragments were identical to the virulent Lausanne sequence. However, each of these samples lacked a single nucleotide within the M138L/M139R intergenic region as was found for the 6918 strain. The 8770 sample (with the wt M135R gene) had the wt M138L/M139R intergenic sequence and also shared a single point mutation within the M148R coding sequence with the Cunivak sample.

**Table 3:** Comparison of insertions/deletions within the nonpathogenic Spanish 6918 myxoma virus strain and Danish clinical samples

		Country of origin/sample						
		ES***^1^	**DK^2^**	**DK**	**DK**	**DK**	**DK**	
	
**Locus**	**Lausanne (wt)**	**Strain 6918 (mut)**	**7889**	**8605**	**8620**	**8773**	**8770**	**Cunivak**
	
M135R	TGTGGGGGTG	TGTGGGGG**G**TG **(+G)**	mut	mut	mut	mut	wt	wt
	
M009L	TCCATCGACATCCA	TC-CA **(-CATCGACATC)**	wt	wt	wt	wt	wt	n.d.*
	
M036L	ACCCCCAGT	ACCCCC**C**AGT **(+C)**	wt	wt	wt	wt	wt	n.d.*
	
M148R	ACCCCCCTTC	ACCC-CCTTC **(-C)**	wt	wt	wt	wt	wt **	wt**
	
M138L/M139R	ATTTTTTTTGTG	ATTT-TTTTGTG **(-T)**	mut	mut	mut	mut	wt	wt
	
M153R/M154R	CTTTTTTTTAAC	CTTT-TTTTAAC **(-T)**	wt	wt	wt	wt	wt	mut

* n.d. = not determined since no PCR product was obtained

** two separate single point mutations within this coding sequence were observed, one of which was common to the sequences of the Cunivak and 8770 samples.

*** data from [14]

1: ES = Spain

2: DK = Denmark

## Discussion

A rapid, sensitive and specific quantitative PCR assay has been developed for myxoma virus which complements previously described qPCR assays for orthopoxviruses (e.g. vaccinia virus, [15]) and parapoxviruses (e.g. Orf virus, [16]). This assay has been shown to function on tissue culture grown viruses and also directly on material extracted from clinical samples. It removes the requirement for specific antisera (which can be variable) or an electron microscope.

Using this assay for myxoma virus, we tested samples from Danish rabbits with clinically determined myxomatosis and we confirmed the diagnosis of the unusually large number of cases of myxomatosis in Denmark during 2007 (see Table 1). The cases were predominantly located in the eastern part of Denmark around Copenhagen, however a small number of cases were also observed elsewhere (as in most previous years). Unexpectedly, a specific frame-shift mutation was present within the coding sequence of the M135R gene in most of the current Danish samples. The insertion of a single additional G, into a run of 5 G's, was present in 10 out of 13 samples analysed. This was the only sequence difference in the M135R gene between each of these samples and the myxoma virus vaccine (which is also identical in this gene to the pathogenic Lausanne strain, [4]). The same nucleotide insertion was also detected in subsequent, independent, PCR reactions which used a different primer set. The generation of these amplicons enabled the fusion of the various M135R gene sequences to the coding sequence of the CAT gene (lacking a termination codon). Expression of these fusion gene plasmids within mammalian cells, in transient expression assays, provided clear evidence that the frame-shift mutation, as detected by the DNA sequencing, indeed resulted in the synthesis of a greatly truncated protein from the mutant M135R sequence (Figure 3).

The M135R gene has recently been described as a virulence factor in myxoma virus [8] since targeted deletion (removing 86% of the coding region) of the gene resulted in severe attenuation of the virus within European rabbits. Experimental inoculation of rabbits with the modified virus gave rise to only moderate symptoms and all animals survived whereas all animals receiving viruses with the intact M135R gene developed severe disease and had to be euthanized. Hence it was surprising that viral DNA obtained from rabbits with clinical disease encoded a greatly truncated version of this gene product comprising only 19 (out of the total 178) amino acids (plus 21 residues translated in a different reading frame). Clearly, the presence of this frame-shift mutation does not severely attenuate the virus within rabbits, since the samples analysed came from animals which had died or were showing significant clinical disease which had lead veterinarians to euthanize the animals. This result is not without precedent. Initial studies, using gene deletion, aimed at determining whether particular poxvirus genes were essential for growth indicated that the vaccinia virus F11L gene was essential for growth [18], however this gene is split in the modified vaccinia Ankara strain indicating that the intact gene is not essential [19]. Subsequent studies using an improved approach demonstrated that this gene is indeed non-essential and showed that misleading results can be obtained from the use of insertional mutagenesis to test for viability [18]. It is, therefore, possible that gene deletion can give misleading data on the requirement of specific genes for virulence. Thus, we conclude from our observations that expression of the full length M135R gene product is not essential for virulence in European rabbits. It would be interesting to assess by transcription profiling (e.g., as described for vaccinia virus [20,21]) whether the targeted deletion of the myxoma virus M135R gene has an influence on the expression of neighbouring genes.

Very recently, Morales et al. [14] reported the complete genome sequence of a nonpathogenic myxoma virus strain (6918) (see [17]) and found that it encoded thirty-two amino acid substitutions plus six insertions or deletions leading to four frame-shift mutations (plus two intergenic region changes) compared to the parental Lausanne strain of the virus (which was 99.95% identical in sequence). One of these frame-shift mutations, within strain 6918, is within the M135R gene and is identical to the change observed within most of the recent Danish samples analysed. It is interesting to note that analysis of recent Portuguese isolates of myxoma virus found evidence of another frame-shift mutation within a virulent strain of this virus which also occurred at a homopolymeric region [22].

To analyse the similarity between the Danish samples and the avirulent 6918 strain, five other regions of the myxoma virus genomes from Danish samples with or without the M135R insertion were amplified by PCR and sequenced. These analyses showed that none of the other frame-shift mutations were present in the Danish samples although the deletion of a single T from within the M138L/M139R intergenic region was a common feature (Table 3). Morales et al. [14] suggested that the frame-shift mutation within the M135R gene may be an important determinant of the attenuation of the 6918 strain since, as described above, deletion of the gene had lead to attenuation [8]. However, the properties of the Danish samples would suggest that some other sequence changes within the 6918 strain are responsible for this phenotype. The critical change(s) could either be amongst the insertions/deletions that are not present in the Danish samples or within the changes leading to the thirty-two different amino acid substitutions.

## Conclusions

1) A sensitive and specific qPCR assay has been established for myxoma virus.

2) An unusually high number of clinical cases of myxoma occurred in Denmark in 2007.

3) A high proportion of the Danish samples that were analysed had a frame-shift mutation within the M135R protein coding sequence which results in the expression of a severely truncated product.

4) The presence of this same frame-shift mutation within an attenuated myxoma virus (6918) is not sufficient to explain its attenuation.

5) Analysis of the effect of the frame-shift mutation within the M135R gene, in isolation, on the virulence of myxoma virus in European rabbits is warranted.

## Competing interests

The authors declare that they have no competing interests.

## Authors' contributions

GJB designed the assay, planned the experiments and drafted the manuscript.

CP performed the transient expression assays and critically reviewed the manuscript.

SØB performed the sequence analysis of the M135R genes and identified the frame-shift mutation within the M135R gene and critically reviewed the manuscript.

LAEL provided the myxoma vaccine, supervised some of the sequencing studies and critically reviewed the manuscript.

ANEB initiated the work, supervised the provision of the clinical samples, had responsibility for the laboratory diagnosis and critically reviewed the manuscript.

All authors read and approved the final manuscript.
